# Effects of VEGF and VEGFR polymorphisms on the outcome of patients with metastatic renal cell carcinoma treated with sunitinib: a systematic review and meta-analysis

**DOI:** 10.18632/oncotarget.19924

**Published:** 2017-08-04

**Authors:** Chenkui Miao, Jingyi Cao, Yuhao Wang, Bianjiang Liu, Zengjun Wang

**Affiliations:** ^1^ State Key Laboratory of Reproductive Medicine and Department of Urology, The First Affiliated Hospital of Nanjing Medical University, Nanjing, China; ^2^ Department of Urology, Xuzhou Cancer Hospital, Xuzhou, China

**Keywords:** VEGF/VEGFR, polymorphisms, metastatic renal cell carcinoma, sunitinib, meta-analysis

## Abstract

To summarize and clarify the association between vascular endothelial growth factor (*VEGF*) and vascular endothelial growth factor receptor (*VEGFR*) polymorphisms and the outcome in patients with metastatic renal cell carcinoma (mRCC) treated with sunitinib. A total of 8 studies including 900 patients were analyzed in this systematic review after screening the database of PubMed, EMBASE and Web of Science. Hazard ratios (HRs) with 95% confidence interval (CI) were used to evaluate the strength of the association. VEGFR1 rs9582036 AA/AC carriers and rs9554320 CC/AC carriers had more favorable overall survival (OS) in patients with mRCC treated with sunitinib (*n* = 3), but not in progression-free survival (PFS). In addition, *VEGFA* rs2010963 was associated with poorer PFS of mRCC (*n* = 1). *VEGFA* rs699947 was significant in predicting PFS by univariate analysis, but showed no statistical significance in OS (*n* = 1). *VEGFR2* rs1870377 was verified to be associated with sunitinib OS (*n* = 1). Furthermore, patients with *VEGFR3* rs307826 and rs307821 had shorter PFS and OS during sunitinib therapy (*n* = 2, respectively). Our results suggested that *VEGF* and *VEGFR* polymorphisms were associated with outcomes in sunitinib treated mRCC patients, especially *VEGFR1* polymorphisms. However, considering the limited study numbers, its clinical application in sunitinib treated mRCC still needs further confirmation.

## INTRODUCTION

Renal cell carcinoma (RCC) is the seventh most common cancer in males and the ninth most common cancer in females, and accounts for nearly 2% of all malignant diseases in adults [1]. Besides, the initial clinical course of RCC is asymptomatic, resulting in 25–30% of patients presenting with metastatic disease when diagnosed [2]. Several molecular-targeted drugs have emerged as the first-line treatment for mRCC patients attributed to its insensitivity to chemotherapy and radiotherapy [3–6]. Sunitinib, as a small-molecule receptor tyrosine kinase inhibitor (TKI), gaining the approval of the Food and Drug Administration (FDA) in 2006. Sunitinib has now been considered as the first-line therapy of mRCC contributed by its anti-angiogenic and anti-tumor activity [7, 8]. Although sunitinib treatment was verified to extend mRCC patients’ survival, accumulating investigations have reported that its effects on outcome might be relevant to specific gene single-nucleotide polymorphisms (SNPs) [9–17]. It has been confirmed that SNPs might connect with pharmacokinetics and pharmacodynamics of sunitinib, thus affecting the prognosis of mRCC patients. However, controversial findings still existed and the conclusion could hardly reach a consensus.

Vascular endothelial growth factor (VEGF) is a potent endothelial cell mitogen that exerts a crucial role in angiogenesis [18, 19]. The VEGF receptors (VEGFR1, VEGFR2 and VEGFR3) also play a significant role in the signaling pathways involved in RCC pathogenesis, and mutations in VEGFRs may affect the signaling networks [20]. Currently, the therapeutic strongholds for metastatic renal cell carcinoma (mRCC) are mostly represented by tyrosine-kinase inhibitors (TKIs) directed against the vascular endothelial growth factor (VEGF) signaling pathway. One of these new molecules, approved for first-line mRCC treatment, is sunitinib [21, 22]. However, previous findings indicated the effective rate was only 60-75%, leaving quite a number of patients to undergo ineffective treatment with added secondary adverse reactions [23].

Therefore, it is really crucial to find appropriate biomarkers closely linked to the clinical outcome in patients with metastatic renal cell carcinoma treated with sunitinib. Recently, several researches focusing on the association between *VEGF* and *VEGFR* polymorphisms and outcome in patients with mRCC treated with sunitinib indicated that some of gene SNPs had significant associations with the survival, while some articles not. Therefore, this systematic review and meta-analysis aimed to conduct an overview of relevant studies, and obtain more comprehensive correlation of *VEGF* and *VEGFR* polymorphisms with the outcome of mRCC patients treated with sunitinib.

## RESULTS

### Characteristics of included studies

8 relevant studies were ultimately enrolled in this systematic review, including 900 patients (635 male and 265 female). Seven eligible studies were performed in Caucasian population except one in Asian. Among the 8 studies, 3 studies reported the correlation of *VEGFA* polymorphisms with patients’ outcome undergoing sunitinib treatment, 3 studies reported *VEGFR1*, 3 focused on *VEGFR2* and 6 investigated *VEGFR3*. In addition, genotyping method, analysis method and metastatic sites were also collected. The detailed summaries of included studies were presented in Table 1. All patients of eligible studies were verified with metastatic RCC and metastases were present in the following organs: lung, liver, bone, brain, lymph nodes, kidney and others. Sunitinib was used as the first line therapy in the treatment of mRCC patients from included investigations. Among these studies, detected gene polymorphisms consisted of *VEGFA* (rs2010963, rs699947, rs1570360), *VEGFR1* (rs9582036, rs9554320), *VEGFR2* (rs1870377) and *VEGFR3* (rs307826, rs448012, rs307821). Besides, the survival data including PFS and OS were extracted from available articles and the following up duration was also recorded. Further information was exhibited entirely in Table 2 and Table 3.

**Table 1:** Main characteristics of included studies in the systematic review and meta-analysis

First author,year	Age	Main Ethnicity	Sample size	Gender		Gene SNPs	Genotyping method	Site of metastasis	Survival analysis	Source of HR	Follow-up time (month)
				Male	Female						
Liu, 2017	62.3 (46–72)	Asian	68	40	28	*VEGFR2/VEGFR3*	PCR-RFLPs	Lung/Lymphatic/Osseous/Hepatic/Adrenal/Other	OS	Reported	median 15 (6–23)
Dornbusch, 2016	59 (53.5–67.0)	Caucasian	121	95	26	*VEGFA/VEGFR1/VEGFR2/VEGFR3*	TaqMan	NA	PFS/OS	Reported	median 24.6 (10.5–41.6)
Beuselinck, 2016	59	Caucasian	157	113	44	*VEGFR1*	Sequenom MassArray platform	Lung/Liver/Bone/Brain	PFS/OS	Reported	median 77 (1–116)
Motzer, 2014	NA	Caucasian	202	135	67	*VEGFR3*	TaqMan	NA	PFS	Reported	NA
Beuselinck, 2014	59	Caucasian	91	62	29	*VEGFR1*	Sequenom MassArray platform	Lung/Liver/Bone/Brain	OS	Reported	median 50 (1–75)
Beuselinck, 2013	59 (38–84)	Caucasian	88	60	28	*VEGFR3*	Sequenom MassArray platform	Lung/Liver/Bone/Brain	PFS/OS	Reported	median 46 (1–73)
Scartozzi, 2013	64 (47–85)	Caucasian	84	65	19	*VEGFA/VEGFR3*	TaqMan	NA	PFS	SC	maximum 42/SC
Garcia-Donas, 2011	65 (42–87)	Caucasian	89	65	24	*VEGFA/VEGFR2/VEGFR3*	KASPar SNP genotyping system	Lung/Lymph nodes/Bone/Kidney/Liver	PFS/OS	Reported	median 21.2 (8.4–25.6)

SNPs, single-nucleotide polymorphisms; *VEGFA*, vascular endothelial growth factor A; *VEGFR*, vascular endothelial growth factor receptor; OS, overall survival; PFS, progression-free survival.

NA, not available; SC, survival curve.

**Table 2:** Association between VEGFA polymorphisms and sunitinib outcome in mRCC

Gene SNPs	First author, year	Allele/Genotype	PFS HR (95% CI)	*P* value	OS HR (95% CI)	*P* value	Analysis method
*VEGFA* rs2010963(G>C)	Dornbusch, 2016	CC+CG vs GG	0.615 (0.357–1.061) M 0.683 (0.463–1.008) U	0.08 M 0.055 U	0.751 (0.354–1.593) M 0.687 (0.403–1.173) U	0.455 M 0.169 U	M/U
	Scartozzi, 2013	CG vs GG	**3.34 (1.19–9.38) U**	**< 0.05 U**	NA	NA	M
	Scartozzi, 2013	CC vs GG	**15.77 (3.11–79.92) U**	**< 0.05U**	NA	NA	M
	Garcia-Donas, 2011	CC vs GG	0.96 (0.62–1.49) M	0.86	1.08 (0.59–1.96) M	0.8	M
*VEGFA* rs699947 (A>C)	Dornbusch, 2016	CC+AC vs AA	1.029 (0.496–2.135) M **0.535 (0.317–0.904) U**	0.939 M **0.019 U**	0.626 (0.256–1.531) M 0.614 (0.316–1.192) U	0.304 M 0.149 U	M/U
	Garcia-Donas, 2011	CC vs AA	1.01 (0.68–1.51) M	0.96 M	0.72 (0.40–1.27) M	0.25 M	M
*VEGFA* rs1570360 (G>A)	Dornbusch, 2016	AA+AG vs GG	0.981 (0.616–1.563) M 1.087 (0.741–1.595) U	0.936 M 0.670 U	0.757 (0.406–1.410) M 0.884 (0.520–1.502) U	0.380 M 0.649 U	M/U
	Garcia-Donas, 2011	AA vs GG	1.13 (0.75–1.70) M	0.56 M	0.79 (0.44–1.44) M	0.44 M	M

The source of HR and 95% CI was extracted from survival curves or article reports.

HRs, hazard ratios; 95% CI, 95% confidence interval; M, multivariate analysis; U, univariate analysis.

SNPs, single-nucleotide polymorphisms; *VEGFA*, vascular endothelial growth factor A; *VEGFR*, vascular endothelial growth factor receptor; OS, overall survival; PFS, progression-free survival.

**Table 3:** Association between VEGFR polymorphisms and sunitinib outcome in mRCC

Gene SNPs	First author, year	Allele/Genotype	PFS HR (95% CI)	*P* value	OS HR (95% CI)	*P* value	Analysis method
*VEGFR1* rs9582036 (A>C)	Dornbusch, 2016	AA+AC vs CC	0.550 (0.197–1.533) M 0.721 (0.362–1.434) U	0.253 M 0.351 U	**0.294 (0.092–0.938)** M **0.294 (0.128–0.676)** U	**0.039** M **0.004** U	M/U
	Beuselinck, 2016	AA+AC vs CC	**0.404 (0.213–0.767)** M **0.25 (0.10–0.63)** U	**0.0056** M **0.003** U	**0.298 (0.159–0.559)** M **0.18 (0.07–0.47)** U	**0.0002** M **0.0004** U	M/U
	Beuselinck, 2014	AA+AC vs CC	NA	NA	**0.2493 (0.07778–0.7992)** M	**0.008** M	M
*VEGFR1* rs9554320 (C>A)	Dornbusch, 2016	CC+AC vs AA	1.454 (0.688–3.070) M 1.107 (0.672–1.823) U	0.327 M 0.690 U	1.233 (0.504–3.015) M 0.959 (0.504–1.825) U	0.646 M 0.899 U	M/U
	Beuselinck, 2016	CC+AC vs AA	**0.486 (0.299–0.787)** M **0.33 (0.18–0.62)** U	**0.0034** M 0.0005 U	**0.488 (0.306–0.775)** M **0.38 (0.21–0.67)** U	**0.0024** M **0.0009** U	M/U
	Beuselinck, 2014	CC+AC vs AA	NA	NA	**0.437 (0.220–0.872)** M	0.067 M **0.019** U	M
*VEGFR2* rs1870377 (T>A)	Liu, 2017	AA vs TT	NA	NA	**3.526 (2.852–5.629)** U	**<0.001** U	U
	Dornbusch, 2016	AA+AT vs TT	1.005 (0.620–1.630) M 0.929 (0.626–1.378) U	0.984 M 0.714 U	0.799 (0.428–1.494) M 0.807 (0.467–1.393) U	0.482 M 0.441 U	M/U
	Garcia-Donas, 2011	AA vs TT	1.09 (0.68–1.74) M	0.71 M	1.74 (0.91–3.32) M	0.092 M	M
*VEGFR3* rs307826 (A>G)	Dornbusch, 2016	GG+GA vs AA	0.460 (0.125–1.694) M 0.645 (0.382–1.088) U	0.243 M 0.100 U	0.907 (0.150–5.481) M 1.245 (0.640–2.419) U	0.915 M 0.519 U	M/U
	Motzer, 2014	GG vs AA	0.94 (0.23–3.81) U	0.929 U	NA	NA	U/NA
	Beuselinck,2013	GG+GA vs AA	1.800 (0.996–3.250) M	0.051 M	**2.223 (1.187–4.163)** M	**0.013** M	M
	Garcia-Donas, 2011	GG vs AA	**3.57 (1.75–7.30)** M	**0.0079** M	1.77 (0.65–4.84) M	0.26 M	M
*VEGFR3* rs448012 (C>G)	Liu, 2017	CC vs GG	NA	NA	**4.113 (3.593–5.942)** U	**< 0.001** U	U
	Garcia-Donas, 2011	GG vs CC	1.12 (0.68–1.85) M	0.66 M	1.36 (0.71–2.59) M	0.35 M	M
*VEGFR3* rs307821 (G>T)	Dornbusch, 2016	TT+TG vs GG	1.351 (0.388–4.707) M 0.722 (0.438–1.190) U	0.636 M 0.201 U	1.349 (0.226–8.066) M 1.239 (0.637–2.408) U	0.743 M 0.528 U	M/U
	Beuselinck, 2013	TT+TG vs GG	**1.981(1.060–3.702)** M	**0.032** M	**2.265(1.202–4.268)** M	**0.011** M	M
	Garcia-Donas, 2011	TT vs GG	**3.31 (1.64–6.68)** M	**0.014** M	1.24 (0.41–3.75) M	0.71 M	M

The source of HR and 95% CI was extracted from survival curves or article reports.

HRs, hazard ratios; 95% CI, 95% confidence interval; M, multivariate analysis; U, univariate analysis.

SNPs, single-nucleotide polymorphisms; *VEGFA*, vascular endothelial growth factor A; *VEGFR*, vascular endothelial growth factor receptor; OS, overall survival; PFS, progression-free survival.

### VEGFA polymorphisms associated with outcome

Three researches involving *VEGFA* rs2010963 are listed in Table 2. In the research conducted by Garcia-Donas et al. in 2011, *VEGFA* rs2010963 polymorphism shows no statistical association with PFS and OS of the mRCC patients received sunitinib, same as that conducted by Dornbusch et al. in 2016 [15, 16]. However, only Scartozzi et al. in 2013 found that PFS proved statistically significance for CG vs GG (HR: 3.34, 95% CI: 1.19–9.38), and CC vs GG (HR: 15.77, 95% CI: 3.11–79.92) (Table 2) [17]. Besides, researches designed by Garcia-Donas et al. in 2011 and Dornbusch et al. in 2016 indicated that *VEGFA* rs699947 was not significantly associated with the PFS and OS of the patients in multivariate analysis [15, 16]. Nevertheless, Dornbusch et al. reported a significant correlation between rs699947 and PFS in univariate analysis (HR: 0.535, 95% CI: 0.317–0.904) (Table 2) [15]. Furthermore, two studies investigating *VEGFA* rs1570360 exerted no obvious results in both PFS and OS of mRCC patients.

### VEGFR1 polymorphisms associated with outcome

**Figure 1:**
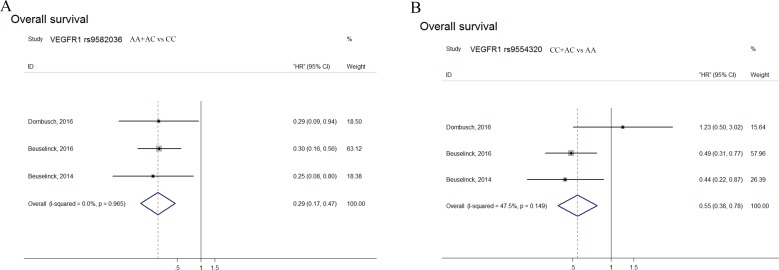
Forest plots of combined analyses associated with *VEGFR1* polymorphisms (**A**): OS with *VEGFR1* rs9582036 (AA+AC vs CC); (**B**): OS with *VEGFR1* rs9554320 (CC+AC vs AA).

Patients with *VEGFR1* rs9582036 AA/AC carriers had superior OS after sunitinib receiving with a pooled HR of 0.29 (95% CI: 0.17–0.47), and rs9554320 with CC/AC carriers also predicted favorable OS (HR: 0.55, 95% CI: 0.38–0.78), but not in patients’ PFS (Figure 1). In addition, Beuselinck's research in 2014 showed that *VEGFR1* rs9582036 had a significant association with the OS of mRCC patients (HR: 0.2493, 95% CI: 0.07778–0.7992) (Table 3) [14]. Beuselinck et al. in 2016 found rs9582036 and rs9554320 was significant in PFS in both multivariate and univariate analysis (Table 3) [13]. What's more, in Beuselinck's study, mRCC patients with the AA-variant in *VEGFR1* rs9554320 have a poorer OS (HR: 2.286, 95% CI: 1.147–4.555) (Table 3). Dornbusch et al., however, did not find any significant association between *VEGFR1* rs9554320 and OS of the patients, PFS either (Table 3) [15].

### VEGFR2 polymorphisms associated with outcome

Among three studies concentrating on effects of *VEGFR2* polymorphism on survival, Liu's study in 2017 reported AA-variant in rs1870377 predicted poorer OS in mRCC patients (HR: 3.526, 95% CI: 2.852–5.629) (Table 3) [24]. Moreover, other two researches failed to found any significant differences (Table 3) [15, 16].

### VEGFR3 polymorphisms associated with outcome

As it came to *VEGFR3* rs307826, there turned up divergences between the different researchers. Four researches concerning this SNP were carried out respectively by Garcia-Donas et al. in 2011, Beuselinck et al. in 2013, Motzer et al. in 2014 and Dornbusch et al. in 2016. Garcia-Donas found that rs307826 was significantly associated with PFS of the patients (HR: 3.57, 95% CI: 1.75–7.30), while other studies not (Table 3) [16]. What's more, Beuselinck's study indicated that there was a significant correlation between rs307826 and OS of the patients with a HR of 2.223 (95% CI: 1.187–4.163), while other researchers found no discrepancy (Table 3) [15, 16, 25, 26]. Furthermore, both Liu et al. and Garcia-Donas et al. studied the relationship of *VEGFR3* rs448012 and the outcome of mRCC patients received sunitinib. They found no statistically significant association between the two in either PFS or OS (Table 3) [16, 24]. As well, data of three studies involving *VEGFR3* rs307821 were also summarized. Garcia-Donas et al. found that rs307821 was associated with PFS of the patients (HR: 3.31, 95% CI: 1.64–6.68), and Beuselinck's research in 2013 arrived at the same result (HR: 1.981, 95% CI: 1.060–3.702) (Table 3) [16, 25]. As regarding the OS, only Beuselinck's study in 2013 indicated a significant association between *VEGFR3* rs307821 and OS of mRCC patients (HR: 2.265, 95% CI: 1.202–4.268), while other two studies found no statistically correlation (Table 3) [15, 16, 25].

### Publication bias

**Figure 2:**
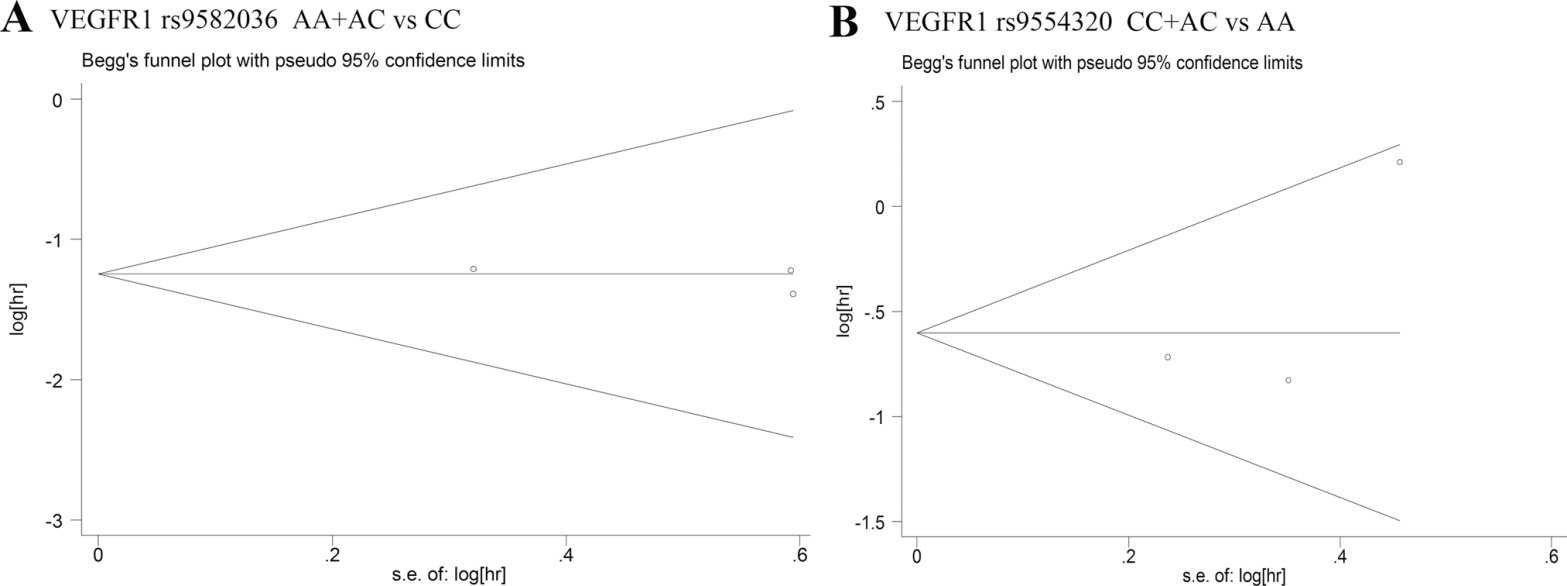
Begg's funnel plots of publication bias test (**A**): OS with *VEGFR1* rs9582036 (AA+AC vs CC); (**B**): OS with *VEGFR1* rs9554320 (CC+AC vs AA).

Begg's test and Egger's test were used to assess the publication bias in this meta-analysis. The Begg's funnel plots with pseudo 95% CIs were symmetric in the pooled analyses (Figures 2A and 2B). *P* values from Egger's test were larger than 0.05, which indicated no obvious publication bias in the meta-analysis.

### Sensitivity analyses

**Figure 3:**
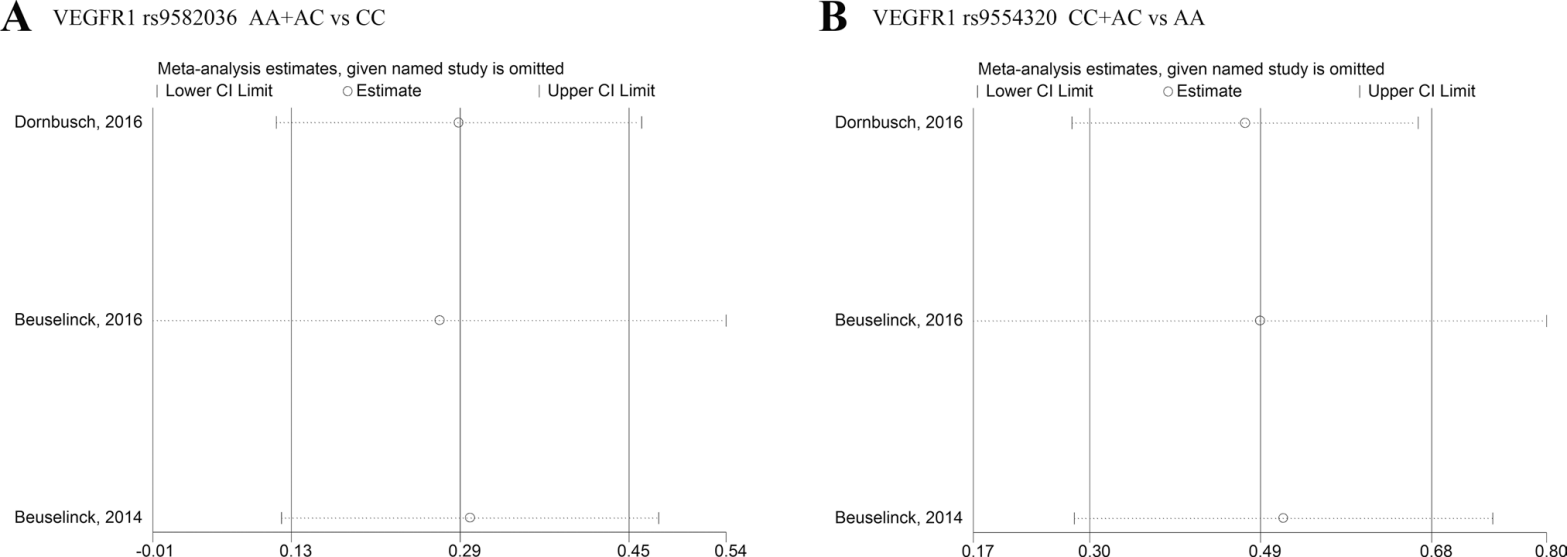
Sensitivity analysis under specific model (**A**): effect of individual studies on the combined HR for OS with *VEGFR1* rs9582036 (AA+AC vs CC); (**B**): effect of individual studies on the combined HR for OS with *VEGFR1* rs9554320 (CC+AC vs AA).

Sensitivity analysis was performed by Stata 12.0 software to evaluated whether individual studies affected the pooled results. Analyses from fixed-effects model indicated that our results are reliable (Figures 3A and 3B).

## DISCUSSION

Sunitinib, as one of orally multiple tyrosine kinase inhibitors (mTKIs), has been approved by FDA in 2006 and widely used as the regular therapy for patients with mRCC [27, 28]. It was suggested that the median PFS period has improved extraordinarily from 5 months with interferon-alpha to 11 months with sunitinib [23]. Approximately up to 47% of RCC patients experienced an objective response and 43% disease stabilization after sunitinib receiving [29]. To a certain degree, targeting angiogenetic pathway with sunitinib could bring about a complete disease alleviation and prolonged mRCC survival. However, during the sunitinib treatment period, PFS and OS of mRCC patients widely ranged from several weeks to years, which might not be easily linked with previous prognostic factors [30]. A growing body of evidence have illuminated that SNPs in several biomarkers including *VEGF*, *VEGFR*, *STAT3* and *interleukin (IL)-8* might be predictive parameters for TKIs therapy [31–33]. Several studies indicated the effects of genetic variability in these biomarkers on sunitinib outcome of mRCC patients might be contributed by the pharmacokinetics and pharmacodynamics of sunitinib. Among these mechanisms, SNPs in *VEGF* pathway owned the most robust clinical evidence, and had been described to involve in sunitinib outcome in mRCC patients [10, 16, 34, 35].

VEGF family consisting of five members: VEGF A, B, C, D and placental growth factor (PLGF), are secreted, dimeric glycoproteins and binding to specific VEGF receptors [36–39]. Studies have suggested that VEGF was aberrantly expressed in RCC, which highlighted that RCC was a VEGF-regulated tumor directly connected with the expression levels of VEGF [40]. Changes in VEGF expression have been reported to be contributed by certain SNPs [30]. Different SNPs in *VEGF* gene may influence its circulating levels, and thus affect its effectiveness response to anti-VEGF therapy. Certain constitutive variation in VEGF and VEGFR expression levels could contribute to a significant difference in RCC outcome during antiangiogenetic treatment. In addition, it was suggested that RCC tumor vasculogenesis has also been associated with the SNPs in *VEGF* and *VEGFR* genes through different biological mechanisms. As a antiangiogenetic drug directly targeting VEGF and VEGFR, sunitinib has been reported to involve in the effect of selected genetic variability on pharmacokinetics and pharmacodynamics in patients with mRCC. SNPs in genes of angiogenesis (VEGFA, VEGFR1, VEGFR2, VEGFR3) as well as gene involved in VEGF-independent pathways were investigated and confirmed to be related to sunitinib metabolism process in mRCC patients [14, 16, 17, 41]. Therefore, different prognosis induced by sunitinib in mRCC patients may be partially ascribed to the vascular pathways mediated by specific *VEGF* and *VEGFR* polymorphisms. Furthermore, accumulating studies have confirmed the association between the survival of patients and the occurrence of SNPs in *VEGF* and *VEGFR* genes, but the results were inconsistent and controversial [13–15]. The predictive role of VEGF and VEGFR polymorphisms in mRCC prognosis remains unclear.

This systematic review and meta-analysis firstly assessed the correlation of *VEGF* and *VEGFR* polymorphisms with sunitinib-treated outcomes in mRCC patients. Sensitivity and heterogeneity analyses were also conducted to evaluate the stability of conclusion of enrolled studies. Our meta-analysis found that AA/AC in *VEGFR1* rs9582036 predicted longer sunitinib OS (HR: 0.29, 95% CI: 0.17–0.47), and rs9554320 with CC/AC carriers was correlated with more favorable OS (HR: 0.55, 95% CI: 0.38–0.78) [13–15]. However, attributed to the limitation of study numbers, the pooled PFS analyses of these two sites were not carried out. So far, since the unified results were obtained between rs9582036, rs9554320 and patients OS with sunitinib, there was no need for subgroup analysis.

In addition, in three studies investigating *VEGFA* polymorphisms, Scartozzi et al. in 2013 found that patients with rs2010963 GG obtained more favorable PFS when compared with CG/CC carriers [17]. Concerning *VEGFA* rs699947 and rs1570360, only Dornbusch's study described a statistically correlation between rs699947 and PFS in univariate analysis but not in multivariate analysis, while others failed to make sense [15–17]. Associations of *VEGFA* polymorphisms in sunitinib outcome were not presented in forest plots by meta-analysis, which was mainly attributed to the limitation of research amounts. Therefore, the results ought to be corrected by further multiple findings. As it came to the effects of *VEGFR2* and *VEGFR3* polymorphisms, only individual studies found their correlation between SNPs and patients prognosis including OS and PFS, which could not be analyzed by statistical methods due to not only the limited investigations, but also differences in genotypes and analyzing models. Thus, associations between *VEGFR2*, *VEGFR3* polymorphisms and mRCC outcomes should be promoted by further evidence.

Admittedly, our systematic review and meta-analysis still has the following deficiencies. Firstly, all studies included were English native, which might contribute to the language bias in results. Secondly, only 8 eligible studies concerning *VEGF* and *VEGFR* polymorphisms and sunitinib outcomes were included in this study, inducing our results might not be adequately persuasive. Further powerful clinical studies are required for more comprehensive conclusion. In addition, only one Asian study was applicable, which might create possible bias to some extent. Regarding these limitations, the potential value of *VEGF* and *VEGFR* polymorphisms in mRCC outcomes might be overly evaluated. Therefore, further large-scale and high-quality studies are needed to verify the associations.

In summary, this study suggested that *VEGF* and *VEGFR* polymorphisms could predict the prognosis of mRCC patients treated with sunitinib, especially *VEGFR1* polymorphisms. Nevertheless, in view of the insufficient data, more subsequent studies on larger cohorts of patients are required to evaluate former findings and to make a validation of the associations between VEGF family and the outcome of sunitinib-treated mRCC patients.

## MATERIALS AND METHODS

### Search strategy

**Figure 4:**
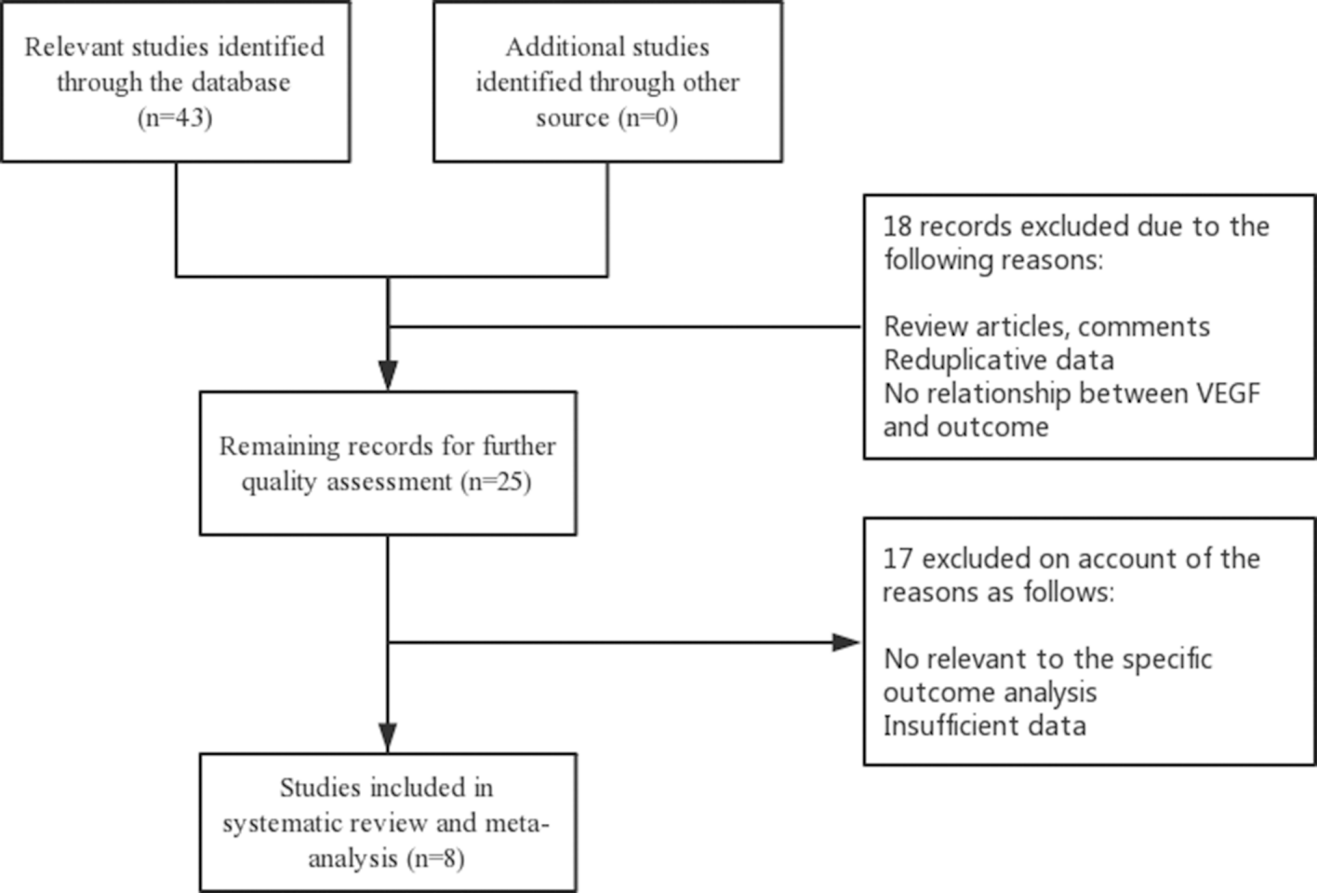
Flow diagram of the study selection process

We searched the relevant papers on PubMed, EMBASE and Web of Science and identified them manually. The keywords we used to search were: “*VEGF* or *VEGFR* or single nucleotide or gene” and “renal cell carcinoma or RCC” and “polymorphism” and “sunitinib”. The last search date was June, 2017. The reference lists of included studies were manually checked for additional publications. A flow diagram of the study selection process is presented in Figure 4.

### Eligibility criteria

Only original articles that focus on the association between *VEGF* and *VEGFR* polymorphisms and outcome in patients with metastatic renal cell carcinoma treated with sunitinib were eligible for our study. Publication language or date was not limited. Additionally, the exclusion criteria were as follows: (1) Studies concentrating on no prognosis in sunitinib treated mRCC. (2) Duplicated data in eligible studies.

### Data extraction

Available data involved in eligible studies were extracted independently by two investigators (Miao CK and Wang YH). The extracted elements were recorded as the following: first author, publication year, main ethnicity, sample size, gender, gene SNPs, genotyping method, site of metastasis, survival analysis, source of HR and follow-up duration. Moreover, the relevant information of association between *VEGF*/*VEGFR* polymorphisms and sunitinib-induced outcome was extracted from enrolled investigations by multivariate or univariate logistic regression analysis. The screened survival indexes consisted of PFS and OS of mRCC patients.

### Statistical analysis

To test the heterogeneity of pooled HRs, Cochran's Q-test and Higgins *I*^2^ statistics (*I*^2^) were performed in the meta-analysis. A fixed-effects model (Mantel-Haenszel method) or random-effects model (DerSimonian-Laird method) was conducted according to the heterogeneity of eligible data. When *P* > 0.05 or the percentage of *I*^2^ was lower than 75%, a fixed-effects model was used to analyze the combined HR, otherwise a random-effects model was utilized. Begg's and Egger's test were utilized to detect the publication bias [42, 43]. Stata 12.0 (Stata Corporation, College Station, TX, USA) was used to calculate all statistical analyses.
